# Metal concentrations in coastal sharks from The Bahamas with a focus on the Caribbean Reef shark

**DOI:** 10.1038/s41598-020-79973-w

**Published:** 2021-01-08

**Authors:** Oliver N. Shipley, Cheng-Shiuan Lee, Nicholas S. Fisher, James K. Sternlicht, Sami Kattan, Erica R. Staaterman, Neil Hammerschlag, Austin J. Gallagher

**Affiliations:** 1 School of Marine and Atmospheric Sciences, Stony Brook University, Stony Brook, NY 11794 USA; 2 New York State Center for Clean Water Technology, Stony Brook University, Stony Brook, NY 11794 USA; 3 Beneath the Waves, PO Box 126, Herndon, VA USA; 4 Rosenstiel School of Marine and Atmospheric Science, University of Miami, Miami, FL 33149 USA

**Keywords:** Biochemistry, Ecology, Physiology, Zoology, Ecology

## Abstract

Over the last century anthropogenic activities have rapidly increased the influx of metals and metalloids entering the marine environment, which can bioaccumulate and biomagnify in marine top consumers. This may elicit sublethal effects on target organisms, having broad implications for human seafood consumers. We provide the first assessment of metal (Cd, Pb, Cr, Mn, Co, Cu, Zn, As, Ag, and THg) and metalloid (As) concentrations in the muscle tissue of coastal sharks from The Bahamas. A total of 36 individual sharks from six species were evaluated, spanning two regions/study areas, with a focus on the Caribbean reef shark (*Carcharhinus perezi*), and to a lesser extent the tiger shark (*Galeocerdo cuvier*). This is due their high relative abundance and ecological significance throughout coastal Bahamian and regional ecosystems. Caribbean reef sharks exhibited some of the highest metal concentrations compared to five other species, and peaks in the concentrations of Pb, Cr, Cu were observed as individuals reached sexual maturity. Observations were attributed to foraging on larger, more piscivorous prey, high longevity, as well a potential slowing rate of growth. We observed correlations between some metals, which are challenging to interpret but may be attributed to trophic level and ambient metal conditions. Our results provide the first account of metal concentrations in Bahamian sharks, suggesting individuals exhibit high concentrations which may potentially cause sublethal effects. Finally, these findings underscore the potential toxicity of shark meat and have significant implications for human consumers.

## Introduction

Over the past century, anthropogenic activities such as rapid industrialization, smelting, and fossil fuel combustion have significantly increased the concentration of metals and metalloids (herein metals) entering marine environments^1^. Many metals (e.g., Cr, Cu, and Zn) are introduced into marine systems via freshwater inputs, effluent run-off, weathering, and ocean–atmosphere interactions^2,3^. Although essential metals such as Cr, Cu, and Zn are required at low concentrations to support healthy cellular processes, many can become toxic when they exceed threshold concentrations^4,5^. These effects may cause sublethal impacts in aquatic organisms such as delayed growth, reproductive impairment, and greater incidence of disease^6,7^ and may have carcinogenic and neurotoxicological impacts for humans^8^. Most metals bioconcentrate and a few biomagnify in marine organisms once they enter the ocean^3,9,10^. Accordingly, long-lived, large-bodied marine predators that exhibit higher trophic positions often display potentially toxic concentrations of metals and other toxicants^11–14^,and can therefore be used as environmental sentinels for regional loadings^15–17^. As many higher trophic-level marine fishes comprise a proportion of the global seafood demand, a need exists to monitor metal concentrations and evaluate the potential toxicity risk for humans readily consuming fish protein^8,16,17^.

Metal concentrations are typically evaluated in higher-order, commercially important fishes to mitigate potential toxic effects on humans; this concern has led to widespread monitoring and scientific study^8,18–20^. However, for higher order predators of historically low commercial value, such as sharks, assessments are much sparser. Sharks are medium to large-bodied predators that occupy meso-to-apex trophic positions throughout marine food-webs^21–23^ and as a result are intrinsic to healthy ecosystem function and resilience^24,25^. Despite the historically low commercial value of shark meat, relative demand as a human protein source appears to be a growing global trend^26^. A need to establish baseline concentrations of metals in sharks and their relatives, as well as potential routes of exposure is therefore required^14,17,20^. This is particularly true for developing nations, where less stringent environmental regulations regarding wastewater treatment, anthropogenic emissions, and subsequent management may lead to elevated levels of metals entering coastal waters.

The developing nation of The Bahamas houses a diversity of productive marine ecosystems such as seagrass beds, oolitic sand banks, open ocean, coral reefs, and mangroves^27^. This high productivity supports biomass of upper trophic level predators, such as sharks, and large teleost fishes^28,29^. High shark diversity and abundance in this region stems from over two decades of legislated protection: commercial long-lining was banned in 1993^30,31^, and the Bahamian EEZ was declared a ‘shark sanctuary’ in 2011, thereby prohibiting the capture, harvest, or trade of shark products within the exclusive economic zone^31,32^. However, the highly migratory nature of coastal sharks^33,34^ may increase fisheries capture and eventual human consumption as a protein source in other neighboring regions of the Greater Caribbean. Baseline monitoring of metal concentrations is therefore necessary to examine the primary route/s of metal exposure and establish the potential toxicity of shark meat. Ultimately, this will allow for the determination of potential hotspots that may benefit from focused environmental management^16^.

This study provides the first assessment of metal (Cd, Pb, Cr, Mn, Co, Cu, Zn, Ag, Hg) and metalloid (As) concentrations in muscle tissue of large-bodied, common shark species from the coastal Bahamas. We present preliminary concentrations for five species: blacknose sharks (*Carcharhinus acronotus*), bull sharks (*Carcharhinus leucas*), tiger sharks (*Galeocerdo cuvier*), nurse sharks (*Ginglymostoma cirratum*), and lemon sharks (*Negaprion brevirostris*). However, we focus much of our analyses on the Caribbean Reef shark *Carcharhinus perezi*^35–37^ owing to their high abundance, ecological significance on Bahamian coral reefs, and high capture rate in neighboring regions where they remain unprotected from fishing and human consumption (e.g., South American fisheries^38^). We also examined correlations between metal concentrations across individuals and examined trends with size. These findings establish the first baseline estimates of metal concentrations in Bahamian sharks with broader implications for the human consumption of shark meat and allow for preliminary inferences regarding the ultimate route/s of metal exposure for these species.

## Results

**Figure 1:**
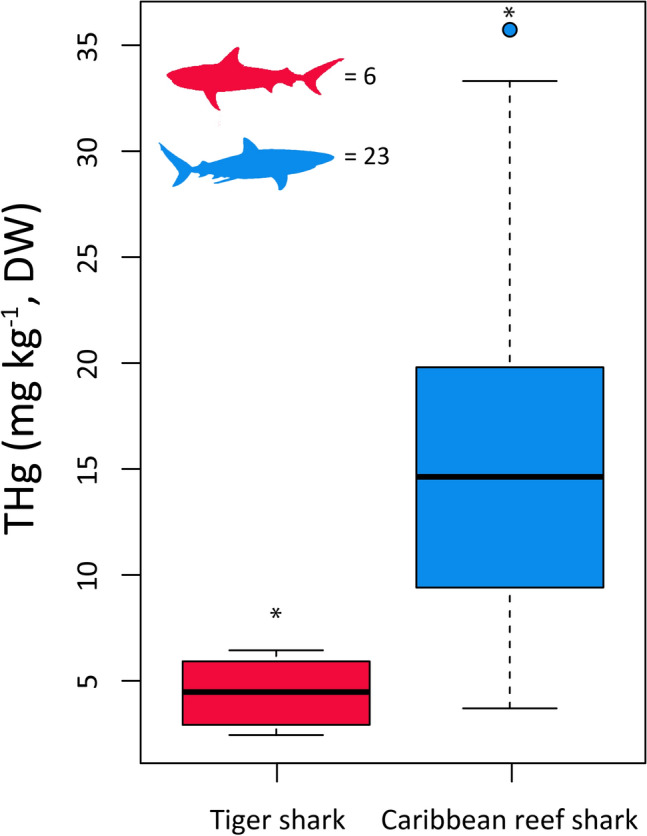
Total mercury concentrations (mg kg^−1^, DW) in the tissues of tiger (*n* = 6) and Caribbean Reef sharks (*n* = 23) sampled from the coastal waters of The Bahamas. Asterisk indicates statistical significance at α = 0.05. The median sizes of sharks sampled was 162 cm for Caribbean reef sharks* and 301 cm for tiger sharks. *Note that median length estimates for Caribbean reef sharks are based off *n* = 22 as a single individual that was measured for THg was DOA and could not be accurately measured for TL.

Metal concentrations in muscle tissue were measured for 36 individuals spanning six species (Table 1). Most sharks sampled were mature based on established size-at-maturity estimates (Compagno et al. 2005), but for Caribbean reef sharks we were able to sample individuals across a broader size range. Total mercury (THg) concentrations were up to 1.5 times higher in Caribbean Reef sharks (16.490 ± 8.331 mg kg^−1^; mean ± SD) than in any other species and these values were higher than those reported in most other sharks species that are commonly found and sampled from The Bahamas and neighboring regions (Table 2). Despite their larger size, tiger sharks exhibited the lowest THg concentrations of all species (4.442 ± 1.619 mg kg^−1^, Table 1), and these results were statistically significant when comparing Caribbean Reef sharks and Tiger sharks (Wilcoxon test, W = 6.000, p < 0.001, Fig. 1).

**Table 1:** Mean heavy metal concentrations (± SD, mg kg^−1^, dw) measured in white muscle tissue of sharks captured from coastal waters of Great Exuma and Nassau New Providence Island, The Bahamas.

Species	Common name	Size range (TL, cm)	*n*	Cd	Pb	Cr	Mn	Co	Cu	Zn	As	Ag	THg
*Carcharhinus acronotus*	Blacknose shark	97–114	3	0.151 (0.028)	0.104 (0.056)^h^	2.577 (2.156)	1.515 (1.396)	0.042 (0.073)	4.227 (1.686)	104.168 (114.608)	2.576 (3.860)	0.067 (0.047)	7.908 (1.747)
*Carcharhinus perezi*	Caribbean reef shark	94–197	24	0.119 (0.085)^e^	0.367 (0.231)^f^	2.641 (3.272)^e^	0.971 (0.659)^e^	0.027 (0.040)^e^	4.897 (3.110)^e^	80.130 (64.833)^e^	7.307 (19.287)^e^	0.066 (0.068)^e^	15.490 (8.331)^g^
*Carcharhinus leucas*	Bull shark	242	1	0.216	0.142	0.09	0.16	0.534	2.73	37	1.38	0.055	6.722
*Galeocerdo cuvier*	Tiger shark	155–320	7	0.138 (0.085)^b^	0.099 (0.074)^c^	0.736 (0.494)^b^	1.765 (1.938)^b^	0.047 (0.033)^b^	3.459 (0.912)^b^	41.298 (7.932)^b^	1.001 (0.764)^b^	0.118 (0.124)^b^	4.442 (1.619)^d^
*Ginglymostoma cirratum*	Nurse shark	204–267	5	0.263 (0.301)	0.108 (0.045)^a^	1.530 (1.112)	0.640 (0.313)	0.009 (0.019)	6.411 (4.412)	88.004 (28.582)	3.750 (3.909)	0.094 (0.126)	9.030 (2.894)
*Negaprion brevirostris*	Lemon shark	240, 248	2	0.231 (0.170)	0.125 (0.108)	2.305 (2.553)	0.467 (0.217)	0.010 (0.014)	30.877 (25.443)	64.140 (37.640)	0.306 (0.432)	0.518 (0.408)	4.846 (0.332)

Sample sizes (*n*) represent the total number of individuals sampled from which metal data were generated, the sample sizes of individual metals may differ based on the removal of data due to potential contamination.

^a^*n* = 4.

^b^*n* = 4.

^c^*n* = 3.

^d^*n* = 6.

^e^*n* = 21.

^f^*n* = 19.

^g^*n* = 23.

^h^*n* = 2.

**Table 2:** Summary of literature-derived total mercury (THg) concentrations (mg kg^−1^, wet weight) reported for shark muscle tissue in species typically found throughout The Bahamas and neighboring regions (updated and adapted from Matulik et al. 2017).

Species	n	Mean	Range/SD	Sampling location	Study
*Carcharhinus limbatus*	21	0.77	0.16–2.3	Florida, US	^39^
*Carchahinus leucas*	53	0.77	0.24–1.7		
*Carcharhinus limbatus*	5	1.9	1.44–2.73	Unknown	^40^
*Carcharhinus spp.*	9	1.61	0.46–4.08	Gulf of Mexico, US	^41^
*Carcharhinus acronotus*	11	1.76	SD: ± 0.8	Florida, US	^12^
*Carcharhinus limbatus*	28	2.65	SD: ± 0.9		
*Carcharhinus leucas*	7	1.48	SD: ± 1.2		
*Nepagrion brevirostris*	2	–	1.67 and 1.69		
*Sphyrna mokkarran*	4	1.65	SD: ± 0.4		
*Galeocerdo cuvier*	8	0.37	SD: ± 0.3		
*Carcharhinus acronotus*	8	2.93	1.65–4.90	Florida, US	^42^
*Carcharhinus leucas*	7	3.95	1.89–7.43		
*Carcharhinus limbatus*	23	3.22	1.20–5.99		
*Nepagrion brevirostris*	8	1.28	0.85–2.40		
*Carcharhinus longimanus*	24	5.04	1.86–11.20	Cat Island, Bahamas	^43^
*Carcharhinus acronotus*	3	2.37	1.84–2.89	New Providence Island and Great Exuma, Bahamas	This study
*Carcharhinus perezi*	24	4.65	1.11–1.72		
*Carcharhinus leucas*	1	2.02	–		
*Galeocerdo cuvier*	7	1.33	0.73–1.93		
*Ginglymostoma cirratum*	5	2.71	1.23–3.54		
*Nepagrion brevirostris*	2	1.45	1.38–1.52		

Concentrations for sharks captured in this study were converted to wet weight by multiplying dry weight concentrations by 0.3 assuming a ~ 70% moisture content reported for shark muscle tissue^44^.

The highest concentrations of Cd were found in the tissues of Nurse sharks (0.263 ± 0.301 mg kg^−1^) and Lemon sharks (0.231 ± 0.170 mg kg^−1^) and the lowest values were found in Caribbean reef sharks (0.119 ± 0.085 mg kg^−1^). The highest concentrations of Pb were found in Caribbean reef sharks (0.367 ± 0.231 mg kg^−1^). For Cr, the highest values were observed in Caribbean reef sharks (2.641 ± 3.272 mg kg^−1^) and blacknose sharks (2.577 ± 2.156 mg kg^−1^). Manganese and cobalt concentrations were highest in blacknose sharks (Mn = 1.515 ± 1.396 mg kg^−1^, Co = 0.042 ± 0.073 mg kg^−1^) and tiger sharks (Mn = 1.765 ± 1.938 mg kg^−1^, Co = 0.047 ± 0.033 mg kg^−1^). Cu concentrations were up to five times higher in lemon sharks (30.877 ± 25.443 mg kg^−1^) than in any other species sampled (< 6.5 mg kg^−1^). Zn concentrations were highest in blacknose sharks (104.168 ± 114.608 mg kg^−1^) and nurse sharks (88.004 ± 28.582 mg kg^−1^). In some species, As concentrations were up to two times higher in Caribbean reef sharks (7.307 ± 19.287 mg kg^−1^) than in the other species sampled (< 9.000 mg kg^−1^). Ag concentrations were over four times higher in lemon sharks (0.518 ± 0.408 mg kg^−1^) than in the other species sampled (< 0.120 mg kg^−1^).

**Figure 2:**
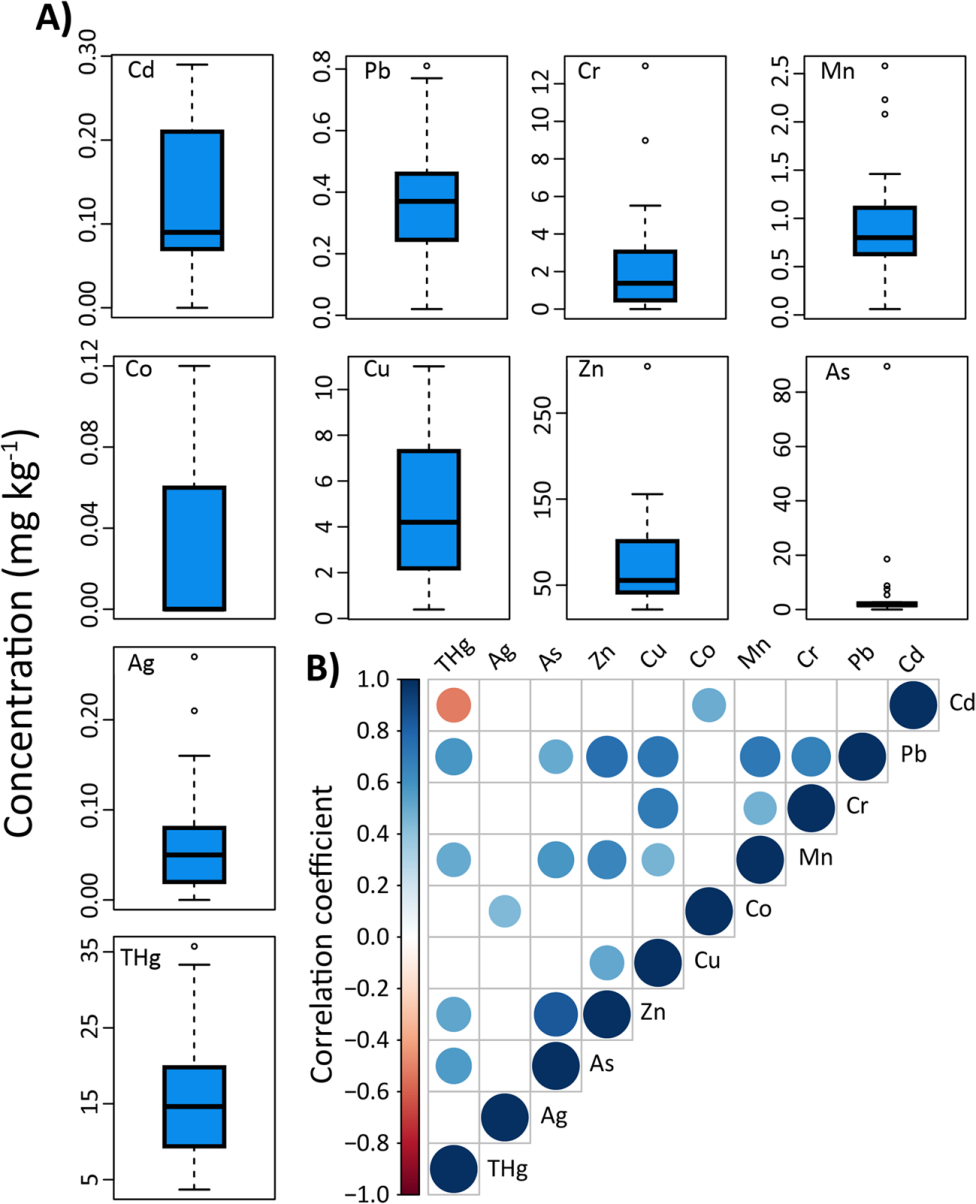
(**A**) Box and whisker plots highlighting the distribution of measurements for metals (mg kg^−1^, DW). Solid horizontal line represents the median, box limits are 1st and 3rd quantiles, whiskers are 1.5 times the interquartile range, and circles represent outliers. (**B**) Correlograms highlighting Spearman’s correlation tests assessing covariance between trace metal concentrations in Caribbean Reef sharks. Circles represent significant correlations at alpha = 0.05 level, size of circles scales with size of correlation (i.e., larger circles indicate higher correlation coefficient) coefficient and colors ramp illustrates whether correlations are positive (blue) or negative (red). See Table 2 for sample sizes associated with each statistical comparison. Correlograms were created in the R package “corrplot” (Wei and Simko, 2017).

We observed strong, positive correlations (r > 0.4) between many of the metals measured within the muscle tissues of Caribbean reef sharks (Table 3, Fig. 2). Cd concentrations were positively correlated with Co, and negatively correlated with THg. Pb concentrations were positively correlated with Cr, Mn, Cu, Zn, As, and THg. Cr concentrations were positively correlated with Mn and Co. Mn concentrations were positively correlated with Cu, Zn, As, and THg. Co concentrations were positively correlated with Ag. Cu concentrations were positively correlated with Zn. Zn concentrations were positively correlated with As and THg. Finally, significant positive correlations were observed between concentrations of As and THg (Table 3, Fig. 2). We observed a negative correlation between THg and Cd.

**Table 3:** Correlation coefficients (p value) for Spearman’s correlation tests examining relationships between trace metal concentrations in the muscle tissue of Caribbean Reef sharks.

	Cd	Pb	Cr	Mn	Co	Cu	Zn	As	Ag	THg
Cd	–	*n* = 19	*n* = 21	*n* = 21	*n* = 21	*n* = 21	*n* = 21	*n* = 21	*n* = 21	*n* = 20
Pb	− 0.076 (0.758)	–	*n* = 19	*n* = 19	*n* = 19	*n* = 19	*n* = 19	*n* = 19	*n* = 19	*n* = 18
Cr	− 0.030 (0.897)	0.664 **(0.002)**	–	*n* = 21	*n* = 21	*n* = 21	*n* = 21	*n* = 21	*n* = 21	*n* = 20
Mn	− 0.187 (0.418)	0.731 **(< 0.001)**	0.661 **(0.001)**	–	*n* = 21	*n* = 21	*n* = 21	*n* = 21	*n* = 21	*n* = 20
Co	0.510 (0.018)	0.323 (0.178)	0.465 **(0.034)**	0.311 (0.170)	–	*n* = 21	*n* = 21	*n* = 21	*n* = 21	*n* = 20
Cu	− 0.076 (0.743)	0.732 **(< 0.001)**	0.768 **(< 0.001)**	0.503 **(0.020)**	0.266 (0.243)	–	*n* = 21	*n* = 21	*n* = 21	*n* = 20
Zn	− 0.141 (0.543)	0.820 **(< 0.001)**	0.487 **(0.025)**	0.548 **(0.010)**	0.110 (0.634)	0.669 **(0.001)**	–	*n* = 21	*n* = 21	*n* = 20
As	− 0.257 (0.261)	0.426 (0.069)	0.244 (0.286)	0.386 (0.084)	− 0.284 (0.213)	0.438 **(0.047)**	0.696 **(< 0.001)**	–	*n* = 21	*n* = 20
Ag	0.060 (0.796)	0.214 (0.379)	0.376 (0.093)	0.003 (0.991)	0.137 (0.553)	0.396 (0.076)	0.194 (0.400)	0.367 (0.102)	–	*n* = 20
THg	− 0.529 (0.016)	0.548 **(0.018)**	0.162 (0.494)	0.457 **(0.043)**	− 0.233 (0.323)	0.334 (0.150)	0.408 (0.075)	0.334 (0.150)	− 0.162 (0.496)	–

Bold indicates statistically significant correlation at α = 0.05 level. Sample sizes for each specific comparison are shown in the second horizontal.

**Figure 3:**
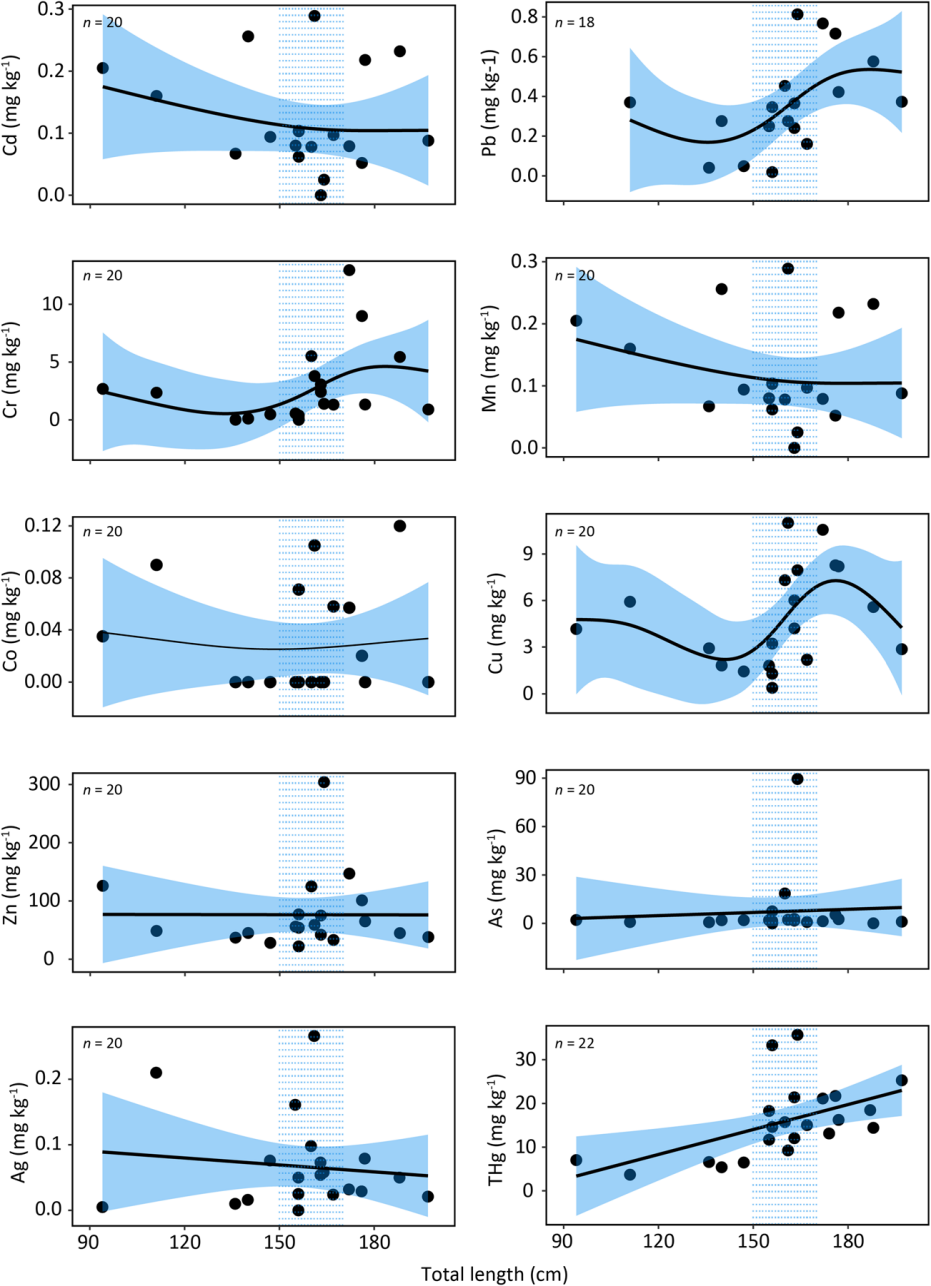
Generalized additive models (GAMs) fit for Caribbean Reef sharks investigating relationships between size and metal concentrations (mg kg^−1^, dw). Blue shaded region represents standard error and blue dotted region represents range of published size-at-maturity estimates (152–168 cm^45^; 150–170 cm, Pikitch et al.^46^). Sample sizes differ between metals owing to removal of data that were potentially contaminated.

Generalized additive models revealed variable trends in metal accumulation with size (Table 4, Fig. 3) for Caribbean reef sharks. For three of the metals (Pb, Cr, and Cu) there appeared to be significant increases in concentrations as individuals approached sexual maturity (152–168 cm^45^; 150–170 cm^46^); a relatively high percentage of the total deviance was also explained by these models (> 39%, Table 4). Trends for Mn, Co, Zn, As, and Ag were less conspicuous, with little or no trend observed. For THg, GAMs revealed a positive, linear relationship with size (Table 4, Fig. 3).

**Table 4:** Deviance explained (%) by generalized additive models between size and metal concentrations for Caribbean Reef sharks.

Metal	Deviance explained (%)
Cd	8.3
Pb	42.6
Cr	39.6
Mn	37.8
Co	4.25
Cu	48.3
Zn	14.4
As	0.6
Ag	30
THg	28.8

## Discussion

This study represents the first evaluation of metal concentrations in large-bodied sharks from The Bahamas. The high and variable concentrations of metals in the muscle tissue of coastal sharks in this region exceeded concentrations considered toxic for human consumption (e.g., THg^8^). Considering the demand for shark meat worldwide^47^, our data provide baseline concentrations of metals and further emphasize the potential toxicity of shark meat for human consumers^8,48,49^. Despite the potential implications for humans, we focus our discussion on the potential drivers of metal concentrations in sharks, and why these may vary across and within taxa. We found that Caribbean reef sharks exhibited the highest concentrations in four of metals (Pb, Cr, As, and THg) relative to other larger-bodied species, some of which peaked as animals approached sexual maturity. We also found some significant (both positive and negative) correlations between metal concentrations in this species, which could be attributed to foraging dynamics, longevity, physiology, and a slower growth rate in older individuals.

A notable finding was the elevated metal concentrations in Caribbean reef sharks, particularly THg, relative to the other larger-bodied species sampled and values reported for other coastal sharks sampled from neighboring regions (see Table 2). Species-specific differences in bioaccumulation trajectories of toxicants have been reported in sharks^12,50^, whereby variable physiologies, trophic ecologies, and maternal offloading may influence the initial concentrations and subsequent bioconcentration^42,51^. One explanation for the generally high metal concentrations in Caribbean reef sharks could be ascribed to a piscivorous diet in larger individuals, foraging upon predominantly larger coral reef-associated fishes (e.g., Grouper, Snapper, and Barracuda), which exhibit high metal concentrations at other Bahamian locales (e.g., South Eleuthera^52^). For THg, the remarkably high concentrations reported here exceed those in nearly all other marine animals^17,53^, which may be ascribed in part to diet^54,55^but also to ambient oceanographic conditions specific to sub-tropical waters such as high ocean temperatures (which increase methylation rates of inorganic Hg by marine microbes^56^). This may explain the higher THg concentrations in less mobile sharks either known or assumed to display high residency within Bahamian waters (e.g., Caribbean reef sharks^36^; blacknose sharks and nurse sharks), relative to more transient species that move throughout much of the northern Western Atlantic ocean, such as tiger sharks^33,34^. Although it remains unknown if high THg concentrations in Caribbean reef sharks elicit neurological effects, there are human health concerns as this species is commonly consumed in certain regions of the Caribbean^57^, as well as in South America^38^. Because THg concentrations were high across multiple species sampled in this study, further evaluation of local effluent and runoff of Hg sources into Bahamian marine systems is certainly warranted. This argument is strengthened by observations of elevated concentrations reaching 0.8 ppm (WW) for THg, which have been observed in teleost species such as king mackerel (*Scomberomorus cavalla*) and great barracuda (*Sphyraena barracuda*) from neighboring waters of South Eleuthera^52^.

Very few studies have explored correlations between metal concentrations in sharks, and trends are often inconsistent among species possibly due to variable metabolisms^14,51,58^. Bosch et al.^8^ found no correlation among metals in smoothound sharks (*Mustelus mustelus*), whereas Kim et al.^59^ found a significant relationship between Hg and Pb in copper sharks (*Carcharhinus brachyurus*). In this study, we found that zinc and manganese were positively correlated with lead, arsenic and mercury in the muscle of Caribbean reef sharks. It is thus possible that these micronutrient metals have some protective effects against heavy metal toxicity^60,61^. Metal competition (e.g., competition for metal binding sites) could lead to negative correlations^62,63^, whereas similar accumulation behaviors, detoxification processes, and similar input sources could result in positive correlations^64,65^. For example, it is suggested that metallothioneins induced by elevated Zn and Cu can interact with and detoxify metal ions such as Cd, Hg, Pb, and Ag^60,61^. Here, we did not observe significant, positive correlations for Zn–Cd and Cu–Cd, but positive correlations were found for Zn–Pb, Zn–Cu, Zn–Hg, and Cu–Pb. Similarly, we found positive correlations between Pb and most of the metals (except Cd, Co, and Ag), implying they might be introduced into the study area though similar geochemical pathways (e.g., dust deposition for Pb and Mn, metal-rich particulate/organic matter from runoff or suspended sediment). Although the ultimate cause and implications of metal correlations are challenging to establish for wild sampled sharks, our descriptive approach indicates that direct experimental study assessing the biological and environmental factors that drive metal correlations, or lack thereof is warranted.

We observed size-based shifts in concentrations of Pb, Cr, Cu, and THg in Caribbean Reef sharks, which peaked as animals reached sexual maturity (Belize: 150–170 cm^46^). Indeed, for Pb, Cr, and Cu. This observation could be explained by a resource-use shift from inshore habitats to deeper continuous reefs^35^, running parallel to deep slopes of the Tongue of the Ocean (near our Nassau, New Providence sampling region) from juvenile/sub-adult to mature life-history stages. This behavior may thus present a different prey base^11^, or ambient concentrations of metals in the water column (i.e., if the primary pathway of metal accumulation is through the gills). As sharks reach sexual maturity, energetic requirements associated with reproduction may increase overall daily energy budgets^44^, requiring individuals to consume a greater biomass of potentially higher trophic position prey items. Although we were unable to definitively test this hypothesis within the confines of this study, ecogeochemical tracer techniques such as stable isotope and fatty acid analyses may provide insight into whether resource-use shifts are in fact occurring between size classes. In other shark species, such as tope sharks (*Galeorhinus galeus*) shifts in metal concentrations have been attributed to habitat shifts^11^, but it is apparent that trends are not uniform across all metals, tissue types, and species. Combined, this suggests that factors other than the organism’s ecology may play a role in the accumulation of metals. For example, metabolic processes, such as reduced growth rates may lead to greater metal accumulation in Caribbean reef shark tissues as processes such as growth dilution are significantly reduced^53^.

## Conclusions

The study provides the first analysis of metal concentrations in the tissues of coastal sharks from The Bahamas. The higher trophic position of the shark assemblage sampled in this study may partly explain why concentrations were elevated. For Caribbean reef sharks, we found the highest levels of harmful THg compared with the other species sampled. We also found peaks in metal concentrations as this species reached sexual maturity, which could be associated with the known ontogenetic shift in habitat/primary prey base combined with growth dilution effects. We recognize that obtaining larger sample sizes should improve comparisons across species, and affirm that there are limitations for interpreting some of the relationships detected here due to knowledge gaps in our understanding of metal trophodynamics in elasmobranch fishes. Overall, our findings suggest that sharks residing within relatively pristine ecological environments may possess high levels of potentially harmful metals, which may have public health implications if they are consumed by local human populations. Further, our findings suggest that Bahamian food-webs may support elevated concentrations of toxic metals and although The Bahamas legally protects sharks from fishing, sublethal impacts may still be induced. As such, future work should seek to determine the habitat-level sources and assimilation factors of metals in sharks and whether overall fitness is affected by high tissue concentrations.

## Methods

### Animal ethics statement

All research was conducted under scientific research permits issued to A. Gallagher (unnumbered) by The Bahamian Department of Marine Resources. Animal handling and sampling protocols followed guidelines listed by the Association for the Study of Animal Behavior^65^. Ethical approval for animal sampling was given by the Canada research chair for animal care (Carelton University, Ottawa, Canada).

### Animal capture and tissue sampling

**Figure 4:**
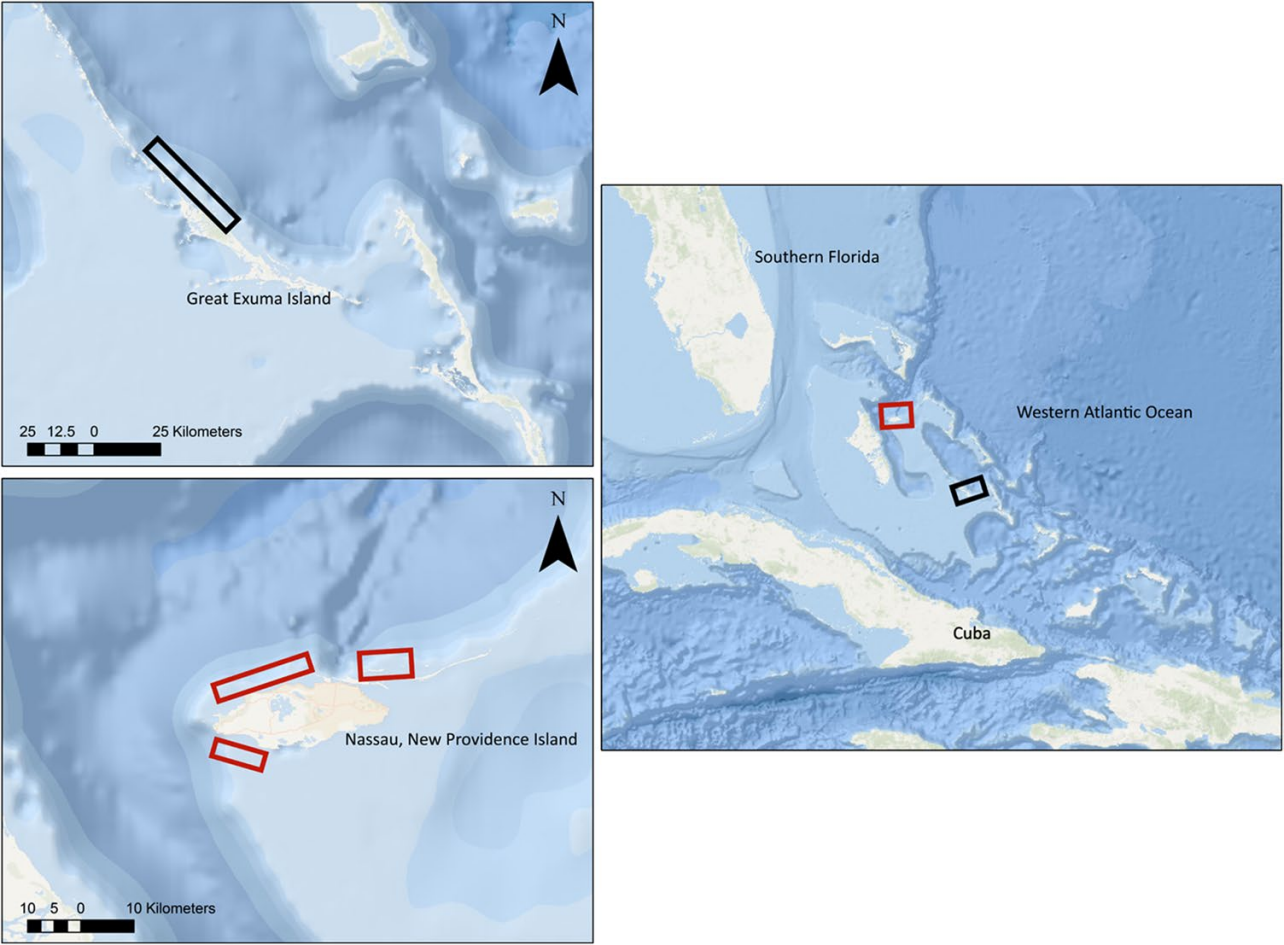
Sampling location of sharks from Great Exuma (black boxes, top left panel) and Nassau New Providence Island (red boxes, bottom left panel), The Bahamas in relation to North Americas and wider western Atlantic Ocean (right panel). Sources: ESRI, GEBCO, NOAA, National Geographic, DeLorme, HERE, Geonames.org, and other.

Sharks were sampled from the coastal waters of Nassau, New Providence and Great Exuma between February 2018 and February 2019 (Fig. 4) using standardized circle-hook research drum lines. Upon capture, animals were secured alongside the research vessel and sex and morphometric measurements were taken. A small incision was made into the dorsal musculature using a sterilized scalpel and approximately 1–2 g of white muscle tissue was excised using a modified 10 mm biopsy punch (Deglon, Thiers, France). All samples were frozen on ice in 2 mL microcentrifuge tubes in the field and then stored − 20 °C before preparation for elemental analysis. Samples were oven dried at 60 °C for ~ 48 h and ground to a fine powder using a mortar and pestle. For statistical purposes, all Caribbean reef shark samples were pooled because capture locations are consistent between islands (e.g., lagoon and forereef habitat), and we cannot discount the movement of individuals between islands.

### Analyses of metals and metalloids

Total mercury (ppm, mg kg^−1^) analysis was conducted on a Milestone DMA-8-Direct Mercury Analyzer. Machine error calculated from repeat measurements of certified reference material (DORM-4) fell within expected ranges (0.412 ± 0.036 mg kg^−1^). The remaining trace metals (Cr, Mn, Co, Cu, Zn, As, Ag, Cd, and Pb) were analyzed by a sector field double focusing high-resolution inductively-coupled plasma mass spectrometer (HR-ICP-MS, Element 2, Thermo Fisher Scientific). Tissue samples were digested into liquid prior to analysis. In brief, a small amount (10 ~ 20 mg) of tissue was soaked in 1 mL of nitric acid (70%, trace metal grade, Fisher Chemical) in a metal-free polypropylene vial at room temperature overnight. After adding 1 mL of hydrogen peroxide (30%, trace analysis, Supelco), the vial was sealed and heated at 90 °C for 12 h until complete digestion. The digest was then combined with Milli-Q water to obtain samples in 2% nitric acid solution which were ready for ICP-MS analysis. ^114^Cd and ^208^Pb were measured in the low-resolution mode, while ^52^Cr, ^55^Mn, ^59^Co, ^63^Cu, ^66^Zn, ^75^As, and ^107^Ag were measured in the medium-resolution mode. Internal standards ^115^In and ^89^Y were used for correcting potential matrix interference. The accuracy of the standard calibration was validated with the certified reference material, Trace Metals in Water Standard A (CRM-TMDW-A, High-Purity Standards). The mid-point standard and the blank were checked every twelve measurements to correct instrumental drifts of the background and the slope of the calibration curve. Two random digest samples were fortified with a known quantity of elements, and the recovery of each spiked element ranged from 74 to 97% (Supplementary Table S1). The certified reference material of fish tissue, DORM-4 (NRCC), was used to ensure the accuracy of the digestion and analytical procedure, which fell within certified ranges (Supplementary Table S2). The instrument detection limit for each metal is listed in Supplementary Table S3. Data above the limit of detection (LOD) were presented. The few samples that exhibited anomalous metal concentrations indicative of contamination were removed from the analyses which are likely to have occurred randomly during subsampling or transportation in the field. The concentration range of each metal presented in this study was comparable to other common shark species reported in earlier literature^8,11^ though the shark species may differ). Note that we reported the concentration on a dry weight basis and the metal concentration would drop 60–80% as converted to wet weight^44^.

### Statistical analyses

All data were analyzed in the statistical programming software R (version 4.0.0). Statistical significance α was 0.05. Shapiro–Wilks tests and F tests were used to examine normality and heteroscedasticity of data, respectively. For Caribbean reef sharks, we examined potential correlation between trace metals through Spearman’s correlation coefficients, presented as correlograms (R package “corrplot”^66^. We used a rank order Spearman’s correlation because some values fell below detection limits and are therefore expressed as zeros. A Wilcoxon signed ranks test was used to examine whether mean THg concentrations statistically differed between Caribbean reef sharks and tiger sharks; low sample sizes for other species and metals precluded comparisons between other species. We also compiled literature derived THg concentrations for shark muscle in species found throughout the Bahamas and neighboring waters for comparative purposes. Because relationships between size and metal concentrations are not necessarily linear, we investigated these using generalized additive models (GAMs, R package “mgcv”^67^). The smoothing parameter k was set to 9 to ensure sufficient degrees of freedom to represent a trend and this parameterization was validated using the ‘gam.check’ function (R package ‘mgcv’) for each fitted model (p > 0.90 for all models^66^).

### Supplementary Information

Supplementary Tables.
