# Supplementary material for: Metal concentrations in coastal sharks from The Bahamas with a focus on the Caribbean Reef shark
Source: Sci Rep. 2021 Jan 8;11:218. doi: 10.1038/s41598-020-79973-w (PMC7794238; doi:10.1038/s41598-020-79973-w)
Supplement: Supplementary file 1 — Supplementary Tables. [file 41598_2020_79973_MOESM1_ESM.docx]

**Metal concentrations in coastal shark species from The Bahamas with a focus on the Caribbean Reef shark**

Oliver N. Shipley^1*^, Cheng-Shiuan Lee^1,2^, Nicholas S. Fisher^1^, James K. Sternlicht^3^, Sami Kattan^3^, Erica Staaterman^3^, Neil Hammerschlag^4^, Austin J. Gallagher^3^

^1^School of Marine and Atmospheric Sciences, Stony Brook University, Stony Brook, NY 11794, USA

^2^New York State Center for Clean Water Technology, Stony Brook University, Stony Brook, NY 11790, USA

^3^Beneath The Waves, PO Box 126, Herndon, VA, USA

^4^Rosenstiel School of Marine and Atmospheric Science, University of Miami, Miami, Florida 33149

**Keywords:** Shark, contaminant, pollution, anthropogenic development, marine protected area, biomagnification, bioaccumulation

[*Corresponding author: Oliver.shipley@stonybrook.edu](mailto:*Corresponding%20author:%20Oliver.shipley@stonybrook.edu)

+1 (631) 215-8619

**Supplementary Information**

Table S1. Fortified sample (1 µg/L spiking) recovery (n = 3).

| ID | Unit | Cd | Pb | Cr | Mn | Co | Cu | Zn | As | Ag |
| --- | --- | --- | --- | --- | --- | --- | --- | --- | --- | --- |
| Mean | % | 84 | 93 | 95 | 93 | 91 | 90 | 75 | 85 | 92 |
| Stdev | % | 1 | 4 | 3 | 1 | 4 | 4 | 3 | 1 | 3 |

Table S2. Measured value vs certified values (n = 6) for DORM-4.

| ID |  | Unit | Cd | Pb | Cr | Mn | Co | Cu | Zn | As | Ag |
| --- | --- | --- | --- | --- | --- | --- | --- | --- | --- | --- | --- |
| Measured value |  | mg/kg | 0.311 | 0.275 | 1.54 | 3.28 | 0.258 | 16.803 | 53.5 | 5.86 | 0.032 |
| Stdev |  | mg/kg | 0.026 | 0.013 | 0.11 | 0.11 | 0.015 | 0.789 | 3.9 | 0.70 | 0.015 |
| Certified value |  | mg/kg | 0.299 | 0.404 | 1.87 | 3.17 | 0.25 | 15.7 | 51.6 | 6.87 | 0.025 |
| Stdev |  | mg/kg | 0.018 | 0.062 | 0.18 | 0.26 | - | 0.46 | 2.8 | 0.44 | 0.005 |
| DORM-4 Recovery |  | % | 104 | 68 | 83 | 104 | 103 | 107 | 104 | 85 | 127 |

Table S3. Detection limits (unit: µg/L) for individual metals analyzed in this study.

|  | | | | |  |  |  |  |
| --- | --- | --- | --- | --- | --- | --- | --- | --- |
| Cd | Pb | Cr | Mn | Co | Cu | Zn | As | Ag |
| 0.009 | 0.003 | 0.017 | 0.005 | 0.006 | 0.017 | 0.023 | 0.100 | 0.004 |
